# Supplementary material for: Wireless battery-free body sensor networks using near-field-enabled clothing
Source: Nat Commun. 2020 Jan 23;11:444. doi: 10.1038/s41467-020-14311-2 (PMC6978350; doi:10.1038/s41467-020-14311-2)
Supplement: Supplementary file 1 — Supplementary Information [file 41467_2020_14311_MOESM1_ESM.pdf]

Supplementary Information for

Wireless battery-free body sensor networks using near-field-enabled clothing

Lin et al.

Supplementary Figures

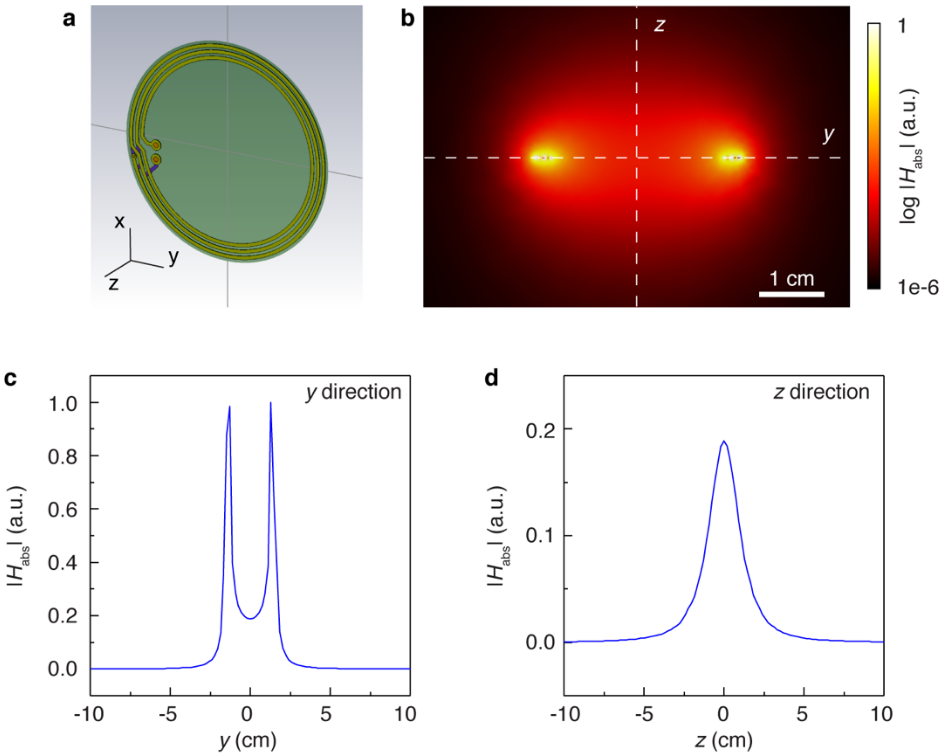

Supplementary Figure 1. Magnetic field distribution emitted by a coil. a, Simulation model of a power source (a 3.1-cm diameter coil). **b**, Normalized magnetic field distribution |H_{abs}| emitted by the coil shown in (a). **c-d**, Field profile along *y* directional dashed line (**c**) and *z* directional dashed line (**d**) in (**b**). The origin is located at the crossing of the two dashed lines.

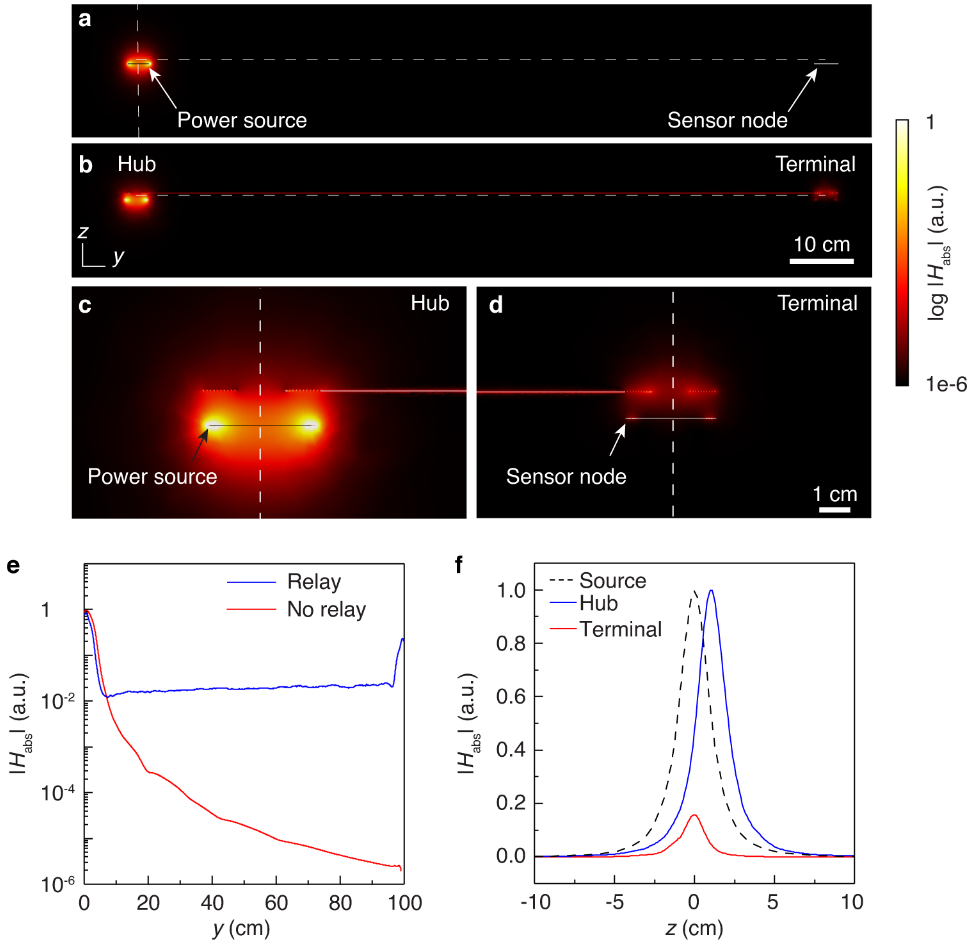

Supplementary Figure 2. Magnetic field distribution around near-field relays. a-b, Normalized magnetic field distribution $|H_{abs}|$ in *y*-*z* plane without (**a**) and with (**b**) a 1-m long near-field relay. The power source and the sensor are 1 m apart in the *y* direction and are positioned 1 cm from the relay in the *z* direction. **c-d**, Normalized magnetic field distribution $|H_{abs}|$ at the hub (**c**) and the terminal (**d**) of the relay. **e**, Field profile in the *y* direction along the horizontal dashed line in (**a-b**). **f**, Field profile in the *z* direction along the vertical dashed line in (**c-d**).

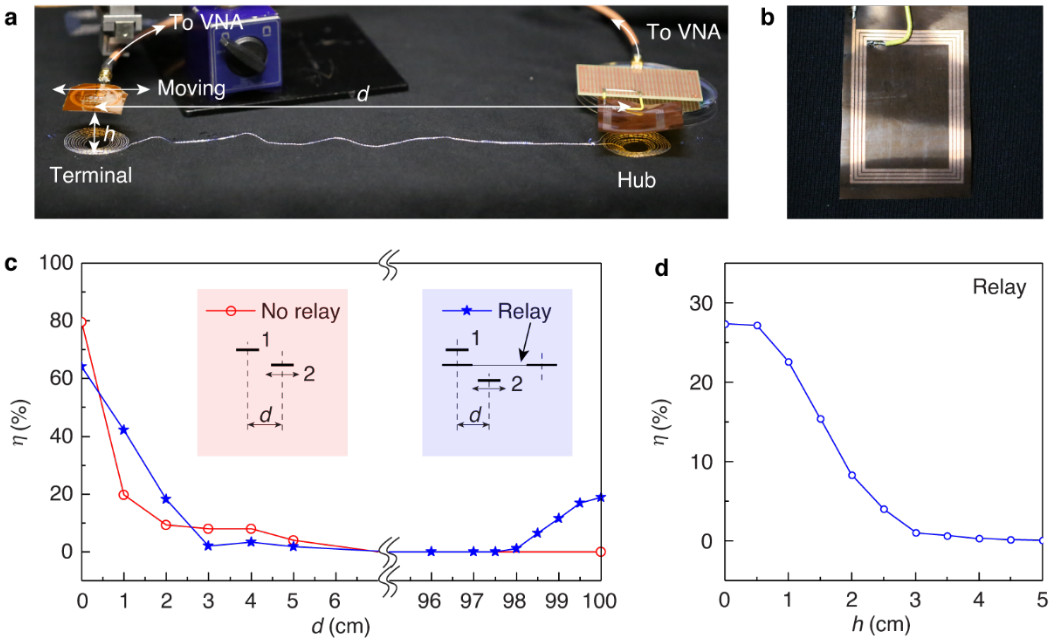

Supplementary Figure 3. Power transfer efficiency from smartphone antenna to sensor. a, Photograph of the experimental setup for measuring the power transfer efficiency η between a smartphone NFC antenna to a sensor node. The near-field relay is 1 m long. *d* is the lateral displacement of the sensor and *h* the vertical distance between the sensor and the relay. **b**, Photograph of the smartphone NFC antenna. The antenna consists of copper on a polyimide substrate and has dimension 3.7 cm × 5.2 cm, wire width of 1mm, and wire gap of 0.3mm. **c**, η as a function of *d* without (in red color) and with (in blue color) the near-field relay for *h*=1 cm. The plot is concatenated in the region *d*=7 to 95 cm where $\eta < 1\%$. **d**, η as a function of vertical distance *h* when the antenna and sensor are concentric to the hub and terminal of the relay.

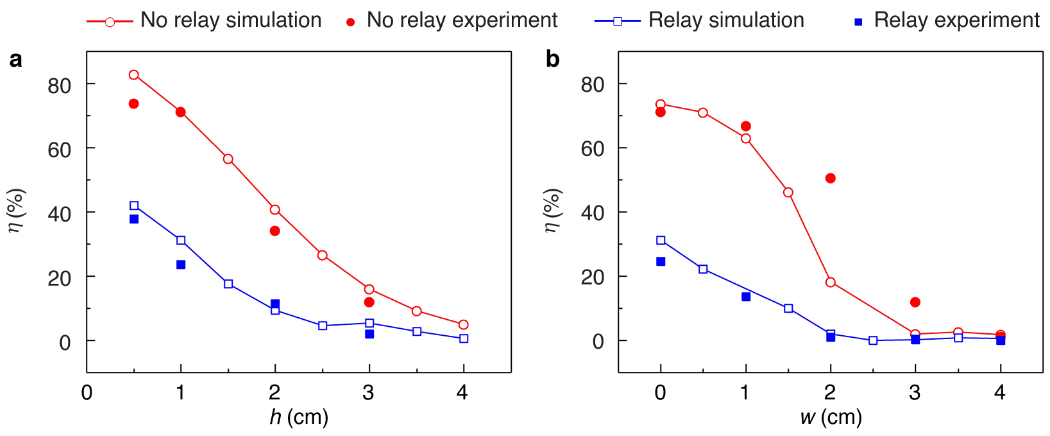

Supplementary Figure 4. Power transferred to a sensor node. a-b, Variation of power transfer efficiency η with moving the senor node away the power source (without relay) or the terminal (with relay) along vertical direction (**a**) and lateral direction (**b**). At the condition of using a 1-m long relay, the reader is placed on top of the hub with 1-cm spacing. When moving along the vertical direction, there is no misalignment $w = 0$ cm. When moving along the lateral direction, the height is fixed at $h = 1$ cm.

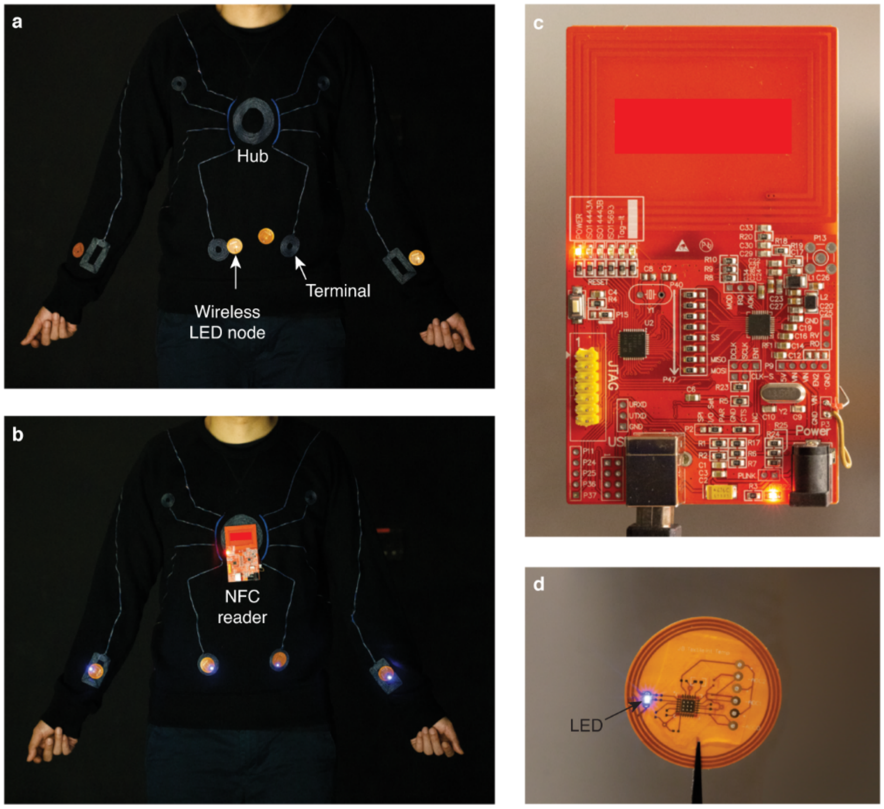

Supplementary Figure 5. Wireless powering of four LED nodes. a, Photograph of a near-field-enabled clothing. The clothing is composed of a hub located at chest and eight terminals distributed around body. Four wireless LED nodes are placed proximity to terminals. **b**, Photograph of wireless powering through the clothing. A battery-powered NFC reader (**c**), which is placed on the central hub, wirelessly powers four LED nodes (**d**) through the near-field-enabled clothing. The LED node is composed of a blue LED connected to a loop antenna with 3.1-cm diameter.

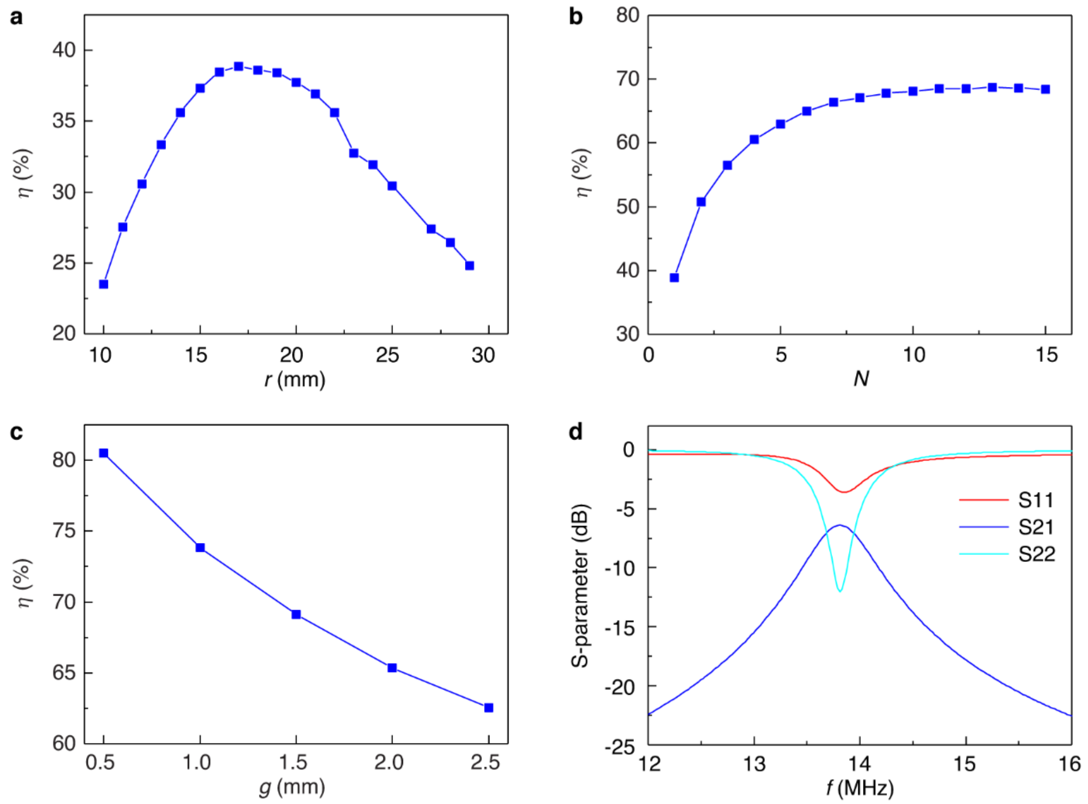

Supplementary Figure 6. Optimization of inductor pattern design. a-c, Dependence of power transfer efficiency η on geometrical parameters of the inductors including (**a**) radius *r*, (**b**) turn number *N*, and (**c**) wire gap *g*. The power is transferred from the power source to the inductor. **d**, Impedance match between the power source and the inductor is achieved at 13.56 MHz.

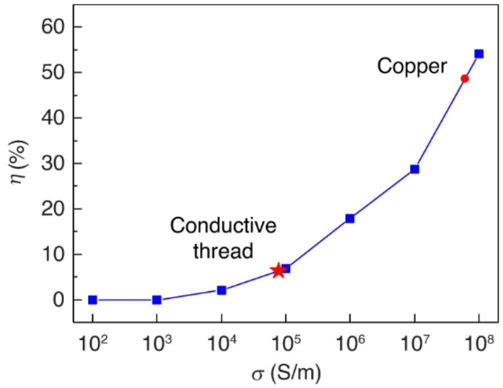

Supplementary Figure 7. Power transfer through near-field relays of varying conductivity. Dependence of power transfer efficiency η on the electrical conductivity σ of the near-field relay. The power is transferred from a wireless reader to a sensor node through a 3-m long near-field relay. The reader and sensor node are respectively placed under the hub and terminal of the relay with 1-cm spacing.

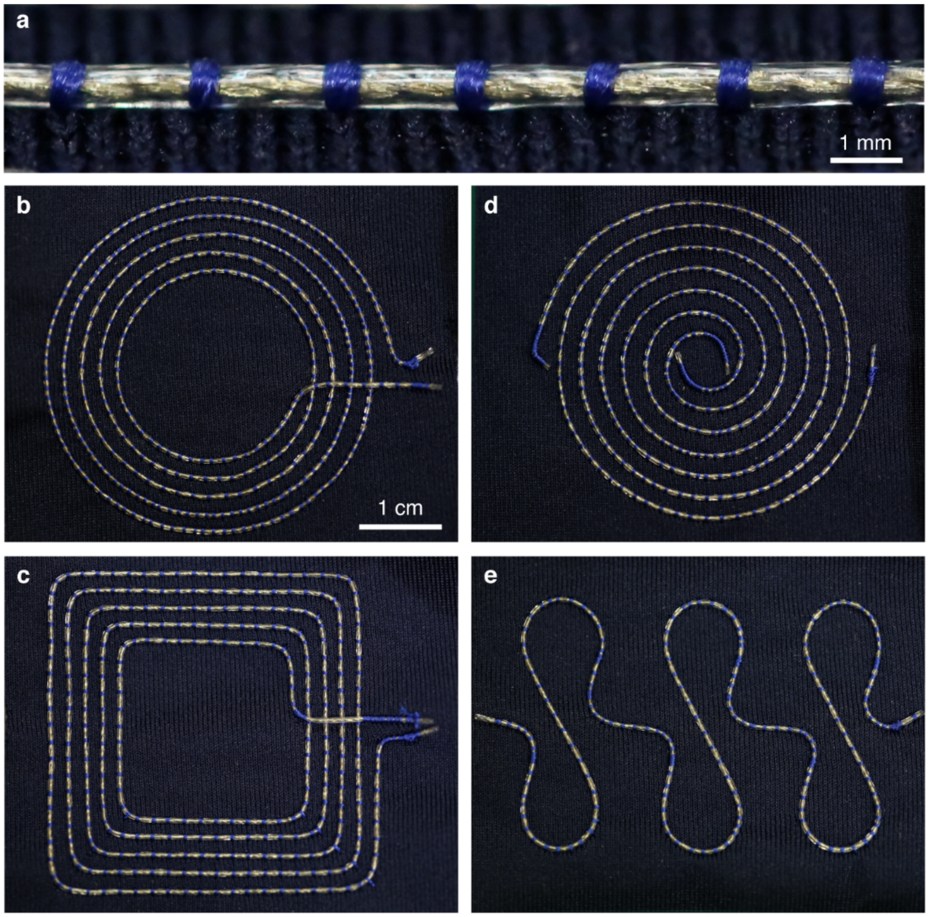

Supplementary Figure 8. Design of pattern. a, A conductive thread is affixed on top of a polyester-spandex clothing by a blue polyester thread. **b-e**, Photographs of conductive thread patterned as a circular spiral coil (**b**), a rectangular spiral coil (**c**), a Archimedes spiral coil (**d**) and a serpentine structure (**e**).

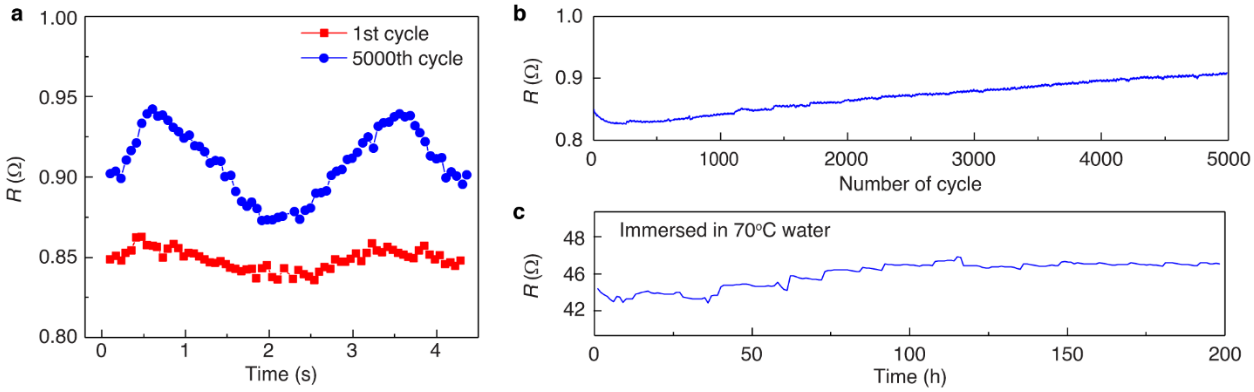

Supplementary Figure 9. Characterization of conductive threads. a, Electrical resistance *R* response of a conductive thread to 1st and 5000th bending cycle (minimum radius 1 mm). **b-c**, Variation of *R* against bending cycle (**b**) and duration of immersion in 70°C hot water (**c**).

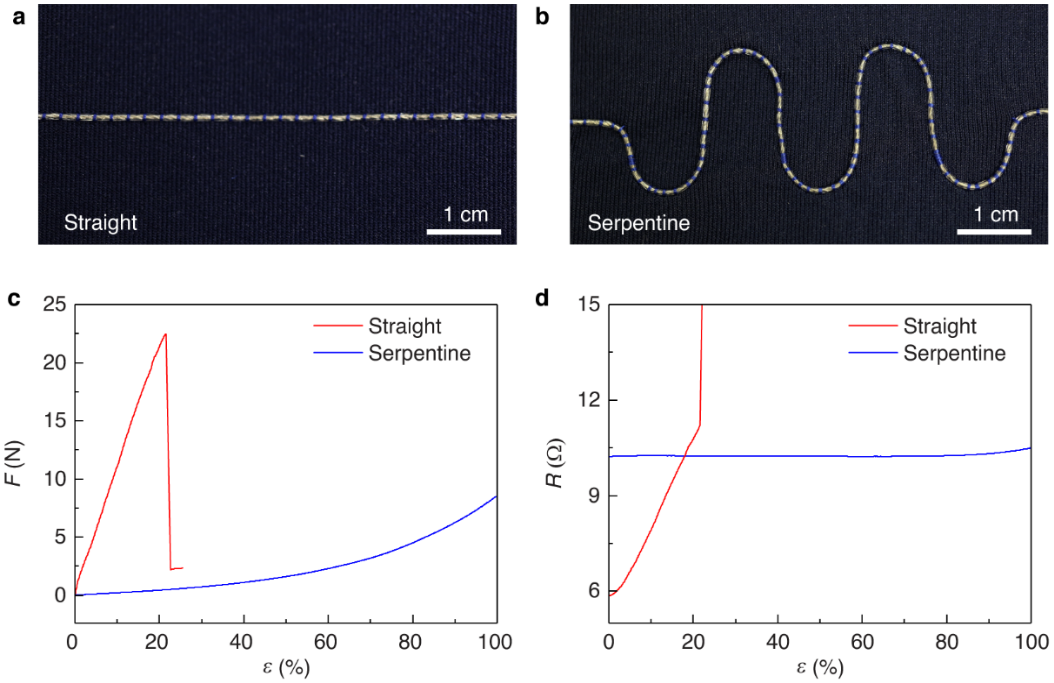

Supplementary Figure 10. Comparison between straight and serpentine structures. a-b, Photographs of a straight structure (**a**) and a serpentine structure (**b**). **c-d**, Dependence of pulling force F (**c**) and electrical resistance R (**d**) on strain ε.

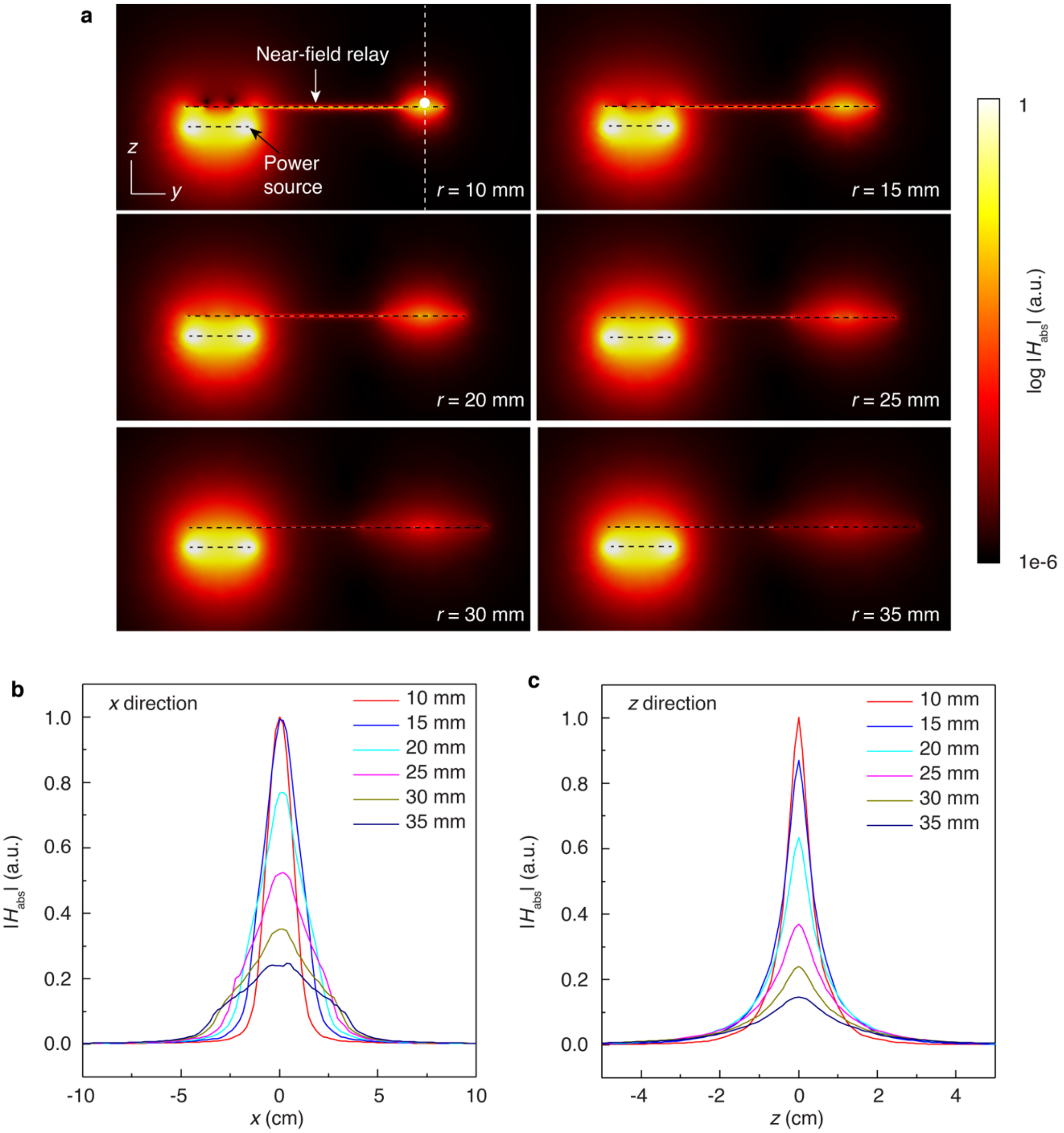

Supplementary Figure 11. Optimization of terminal radius. **a**, Normalized magnetic field distribution $|H_{abs}|$ with varying terminal radius r of the near-field relay. The terminals maintain constant inner radius (2 mm) and wire gap (1 mm). **b-c**, Field profile along (**b**) x direction (white dot in (**a**)) and (**c**) z direction (white dash line in (**a**)).

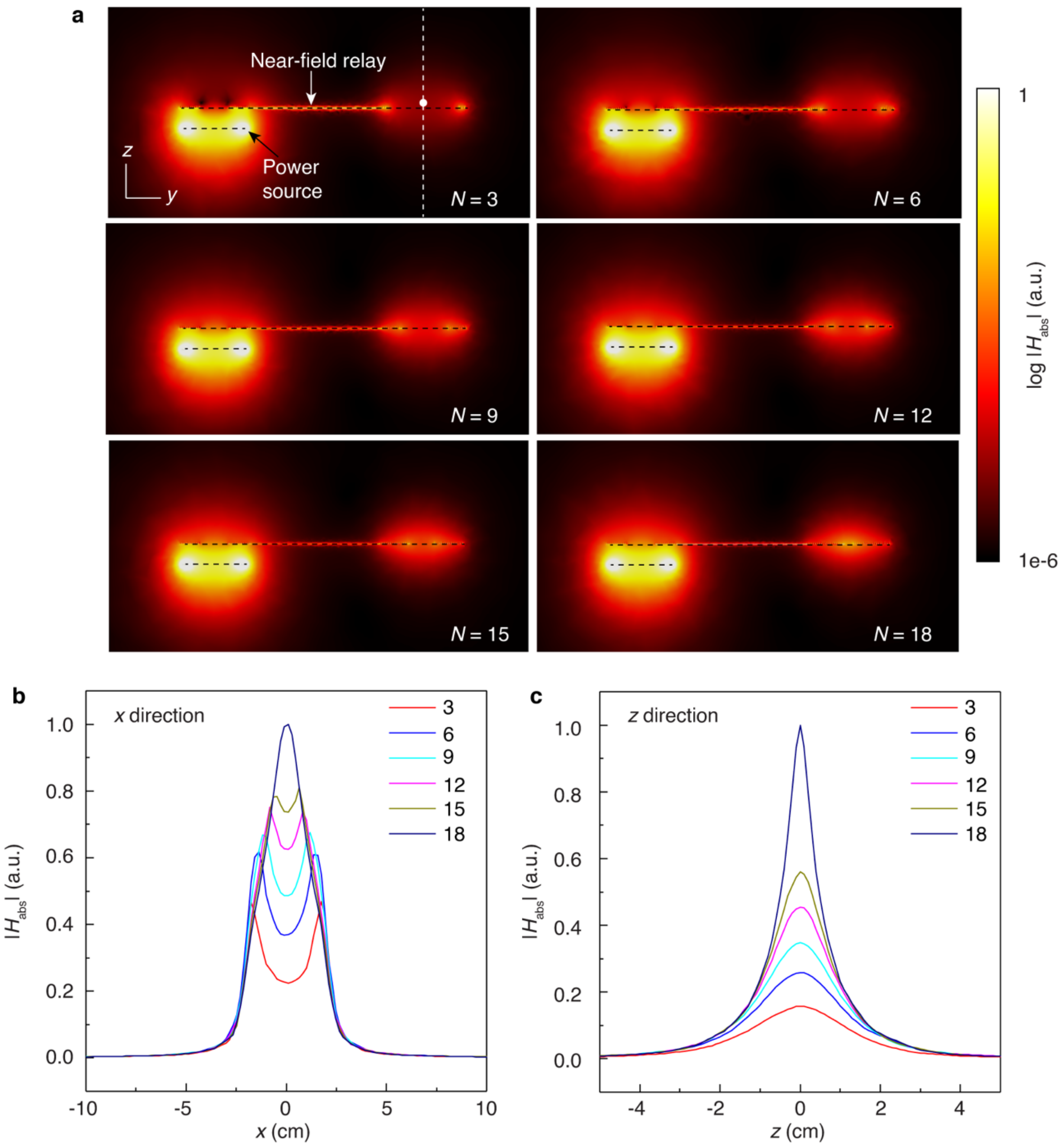

Supplementary Figure 12. Optimization of terminal turn number. a, Normalized magnetic field distribution $|H_{abs}|$ with varying terminal turn number *N*. The terminals maintain constant outer radius (20 mm) and wire gap (1 mm). **b-c**, Field profile along (**b**) *x* direction (white dot in (**a**)) and (**c**) *z* direction (white dash line in (**a**)).

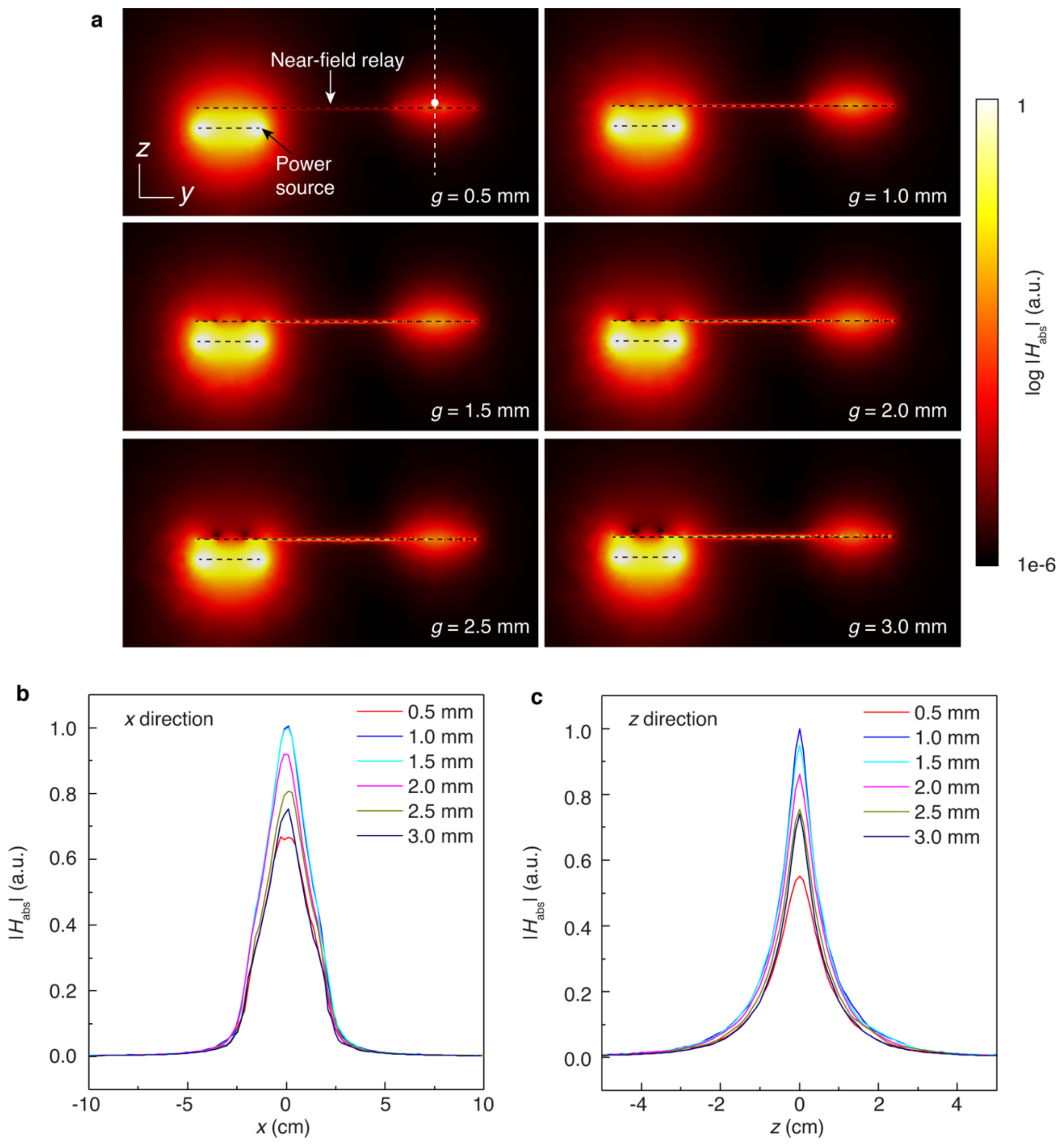

Supplementary Figure 13. Optimization of terminal wire gap. a, Normalized magnetic field distribution |H_{abs}| with varying wire gap *g*. The terminals maintain constant outer radius (20 mm) and inner radius (2 mm). **b-c**, Field profile along (**b**) *x* direction (white dot in (**a**)) and (**c**) *z* direction (white dash line in (**a**)).

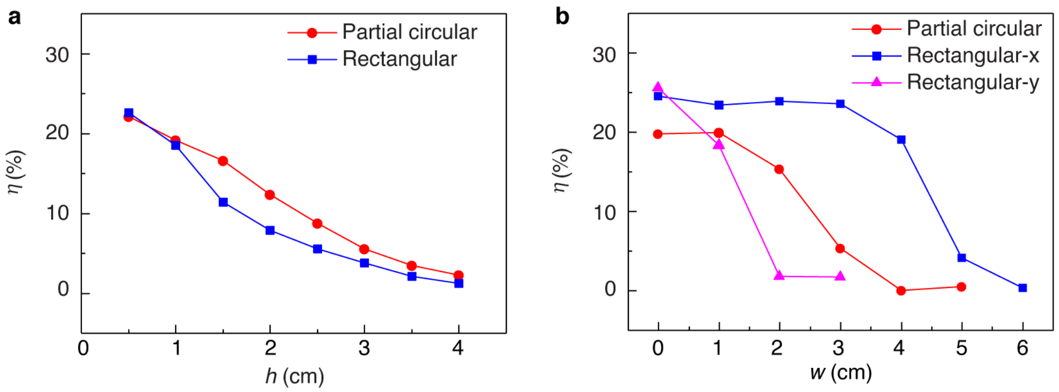

Supplementary Figure 14. Power transferred through optimized near-field relays. a-b, Variation of power transfer efficiency η with moving the senor node away the terminal along vertical direction (**a**) and lateral direction (**b**). Both the partial circular terminal (7.2-cm outer diameter) and rectangular terminal (10 cm × 4 cm dimension) have the same filling area (~25 cm^2) and filling are ratio (~38%). When the sensor node is moved along the vertical direction, there is not misalignment $w = 0$ cm. When the sensor node is moved along the lateral direction, the height is fixed at $h = 1$ cm.

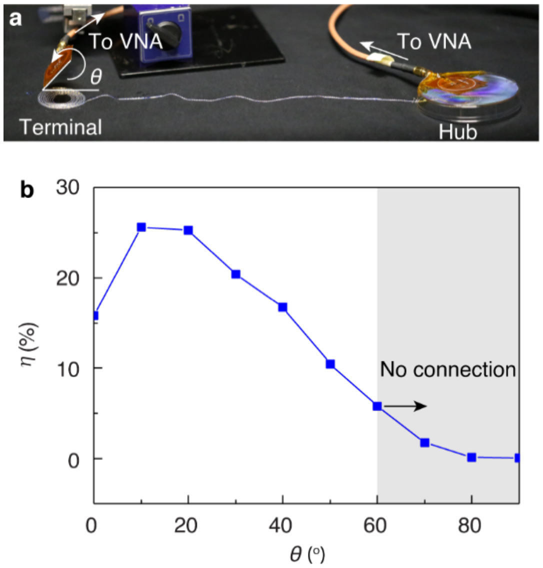

Supplementary Figure 15. Effect of angular misalignment on near field communication. a, Illustration of power transferring from a reader tag to a sensor node via a 1-m long relay. The reader and the sensor node are placed 1cm and 2cm above the relay, respectively. **b**, Dependence of power transfer efficiency η on the angular misalignment Θ between the sensor node and the relay. There is no connection between the NFC reader and the sensor node when $\Theta > 60^{\circ}$.

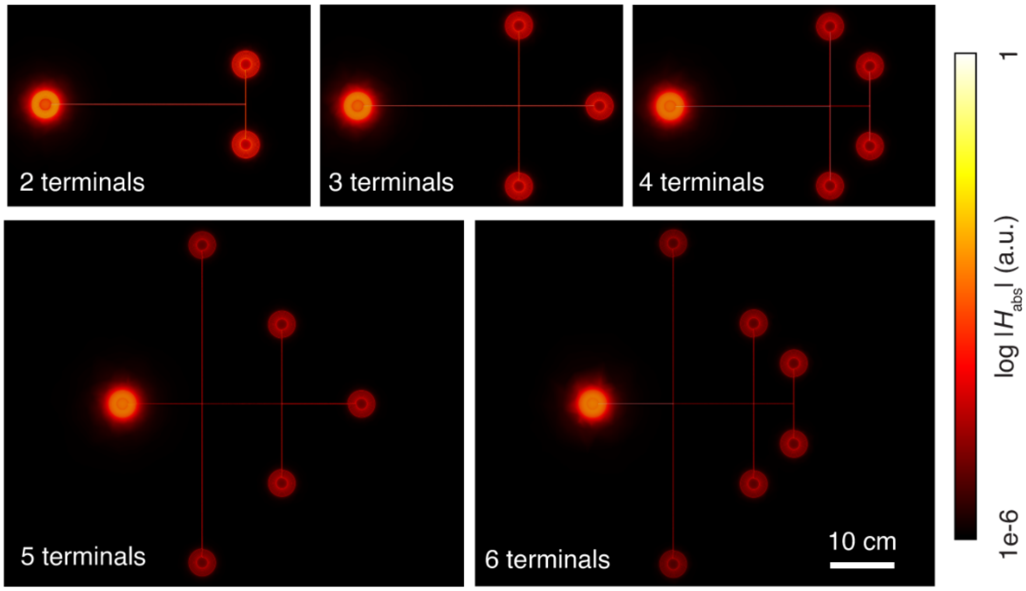

Supplementary Figure 16. Multiterminal near-field relay magnetic field distribution. Normalized magnetic field distribution |H_{abs}| for near-field relays with increasing terminal number from 2 to 6. All terminals are connected to the hub in a series pattern, and the distance from each terminal to the hub is 30 cm. The power source/sensor node is placed on the hub/terminal with 1-cm spacing.

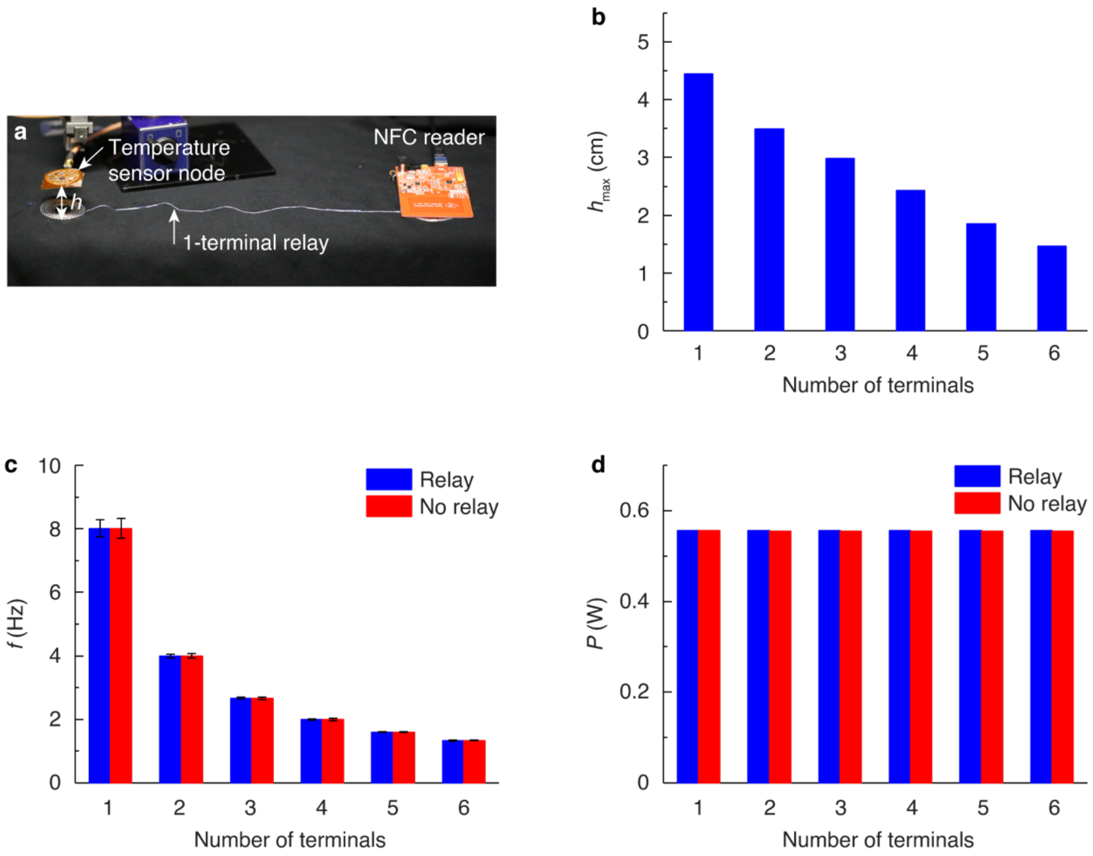

Supplementary Figure 17. Communication performance scaling with number of terminals. **a**, Photograph of the experimental setup with a 1-terminal relay consisting of a NFC reader placed 1 cm above the hub and a temperature sensor placed distance *h* above the terminal. The relay is 30-cm long and the number of terminals in the parallel configuration increased as shown in Fig. 2h. **b,** Maximum vertical distance h_{max} at which NFC connection can be established as a function of number of terminals. **c,** Sampling frequency *f* of each sensor by the reader as a function of number of terminals. Error bars show mean ± s.d. (*n*=100 samples). **d,** Total power consumption *P* of the reader a function of number of terminals. The antenna output power is set to 200 mW.

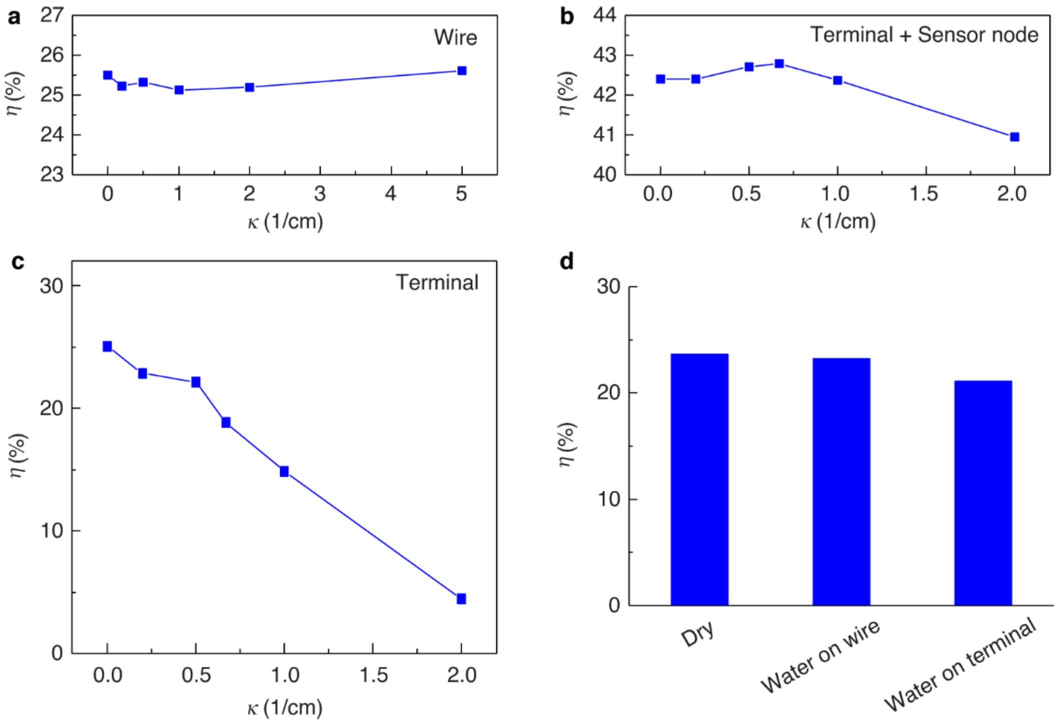

Supplementary Figure 18. Power transfer during bending and wetting of near-field relays. **a-c,** Dependence of power transfer efficiency η on the bending curvature κ for bending wire (**a**), both terminal and sensor node (**b**), and terminal only (**c**). The minimum spacing between the sensor node and the terminal is 1 cm when bending wire or terminal, while the sensor node is attached to the terminal when bending both of them simultaneously. **d**, Dependence of η on water splashing.

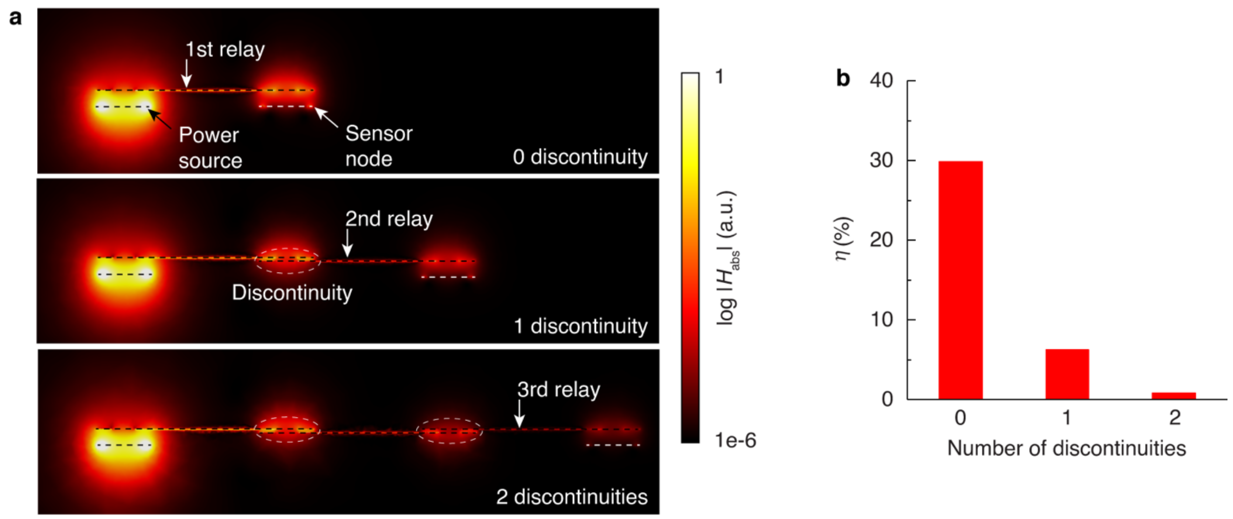

Supplementary Figure 19. Power transferred across discontinuities. a, Normalized magnetic field distribution |H_{abs}| across discontinuities. The spacing between neighboring relays is 2 mm. **b**, Dependence of power transfer efficiency η on the number of discontinuities.

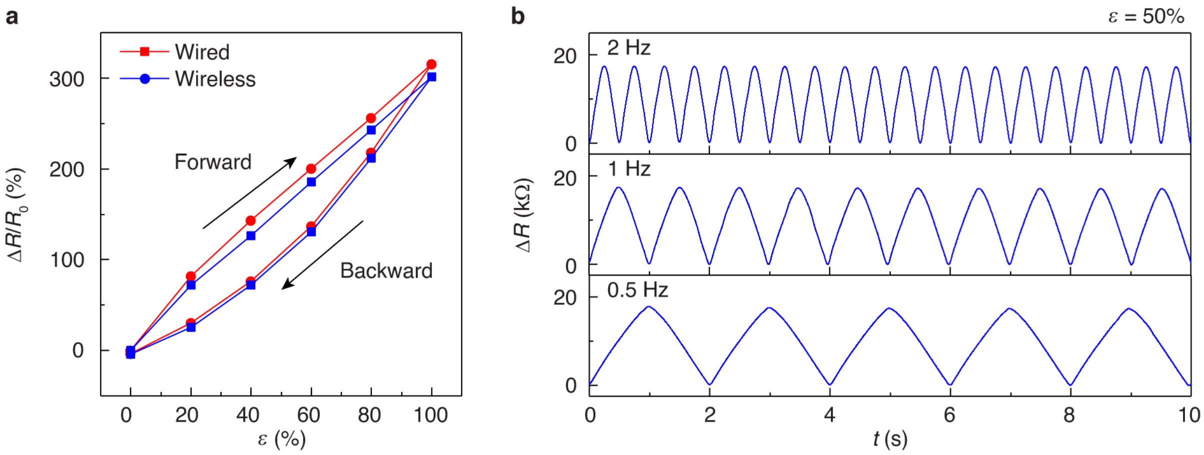

Supplementary Figure 20. Calibration of strain sensors. a, Dependence of relative resistance change $\Delta R/R_0$ on strain $\mathcal{E}$ cycled from 0 to 100% with a step of 20%. The same strain sensor is connected in a wired approach (to a digit multimeter) or wireless approach (to a NFC tag). **b**, Response of a strain sensor to $\mathcal{E}$ = 50% at applied frequency of 0.5 Hz, 1 Hz and 2 Hz.

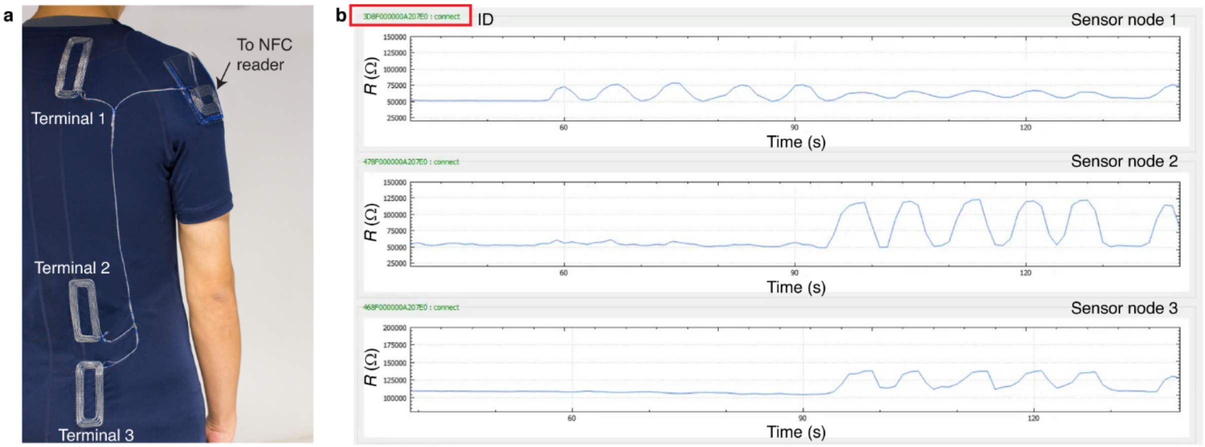

Supplementary Figure 21. Multi-node spine posture monitoring. a, Photograph of a near-field-enabled clothing with a hub located at the sleeve and three terminals distributed along cervical, thoracic and lumbar spine. A transparent and flexible plastic pocket is sewed on top of the hub for holding the NFC reader. Three battery-free wireless sensor nodes are attached to the skin under each terminal. **b**, Screenshot of computer application displaying data received from three wireless sensor nodes on the spine (sensor node 1 to 3) via the near-field-enabled clothing.

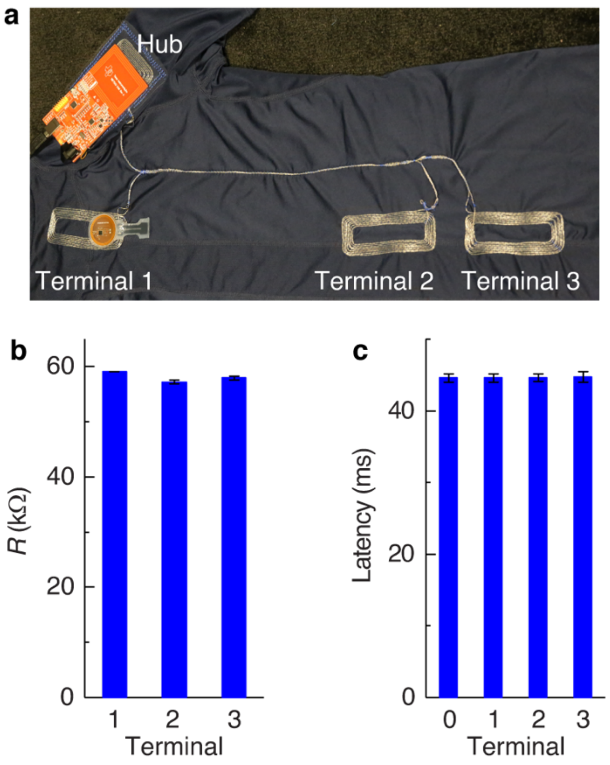

Supplementary Figure 22. Variation in sensor readout from different terminals. a, Photograph of the experimental setup consisting of a NFC reader, a strain sensor node, and the relay network shown in Fig. 3 (hub and three terminals along the spinal column). **b-c**, Strain sensor resistance *R* (**b**) and latency of the wireless measurement (**c**) wirelessly measured at each terminal position. Terminal 0 represents close proximity wireless connection to the hub without the relay. Error bars represent mean ± s.d. (*n*=100 measurements).

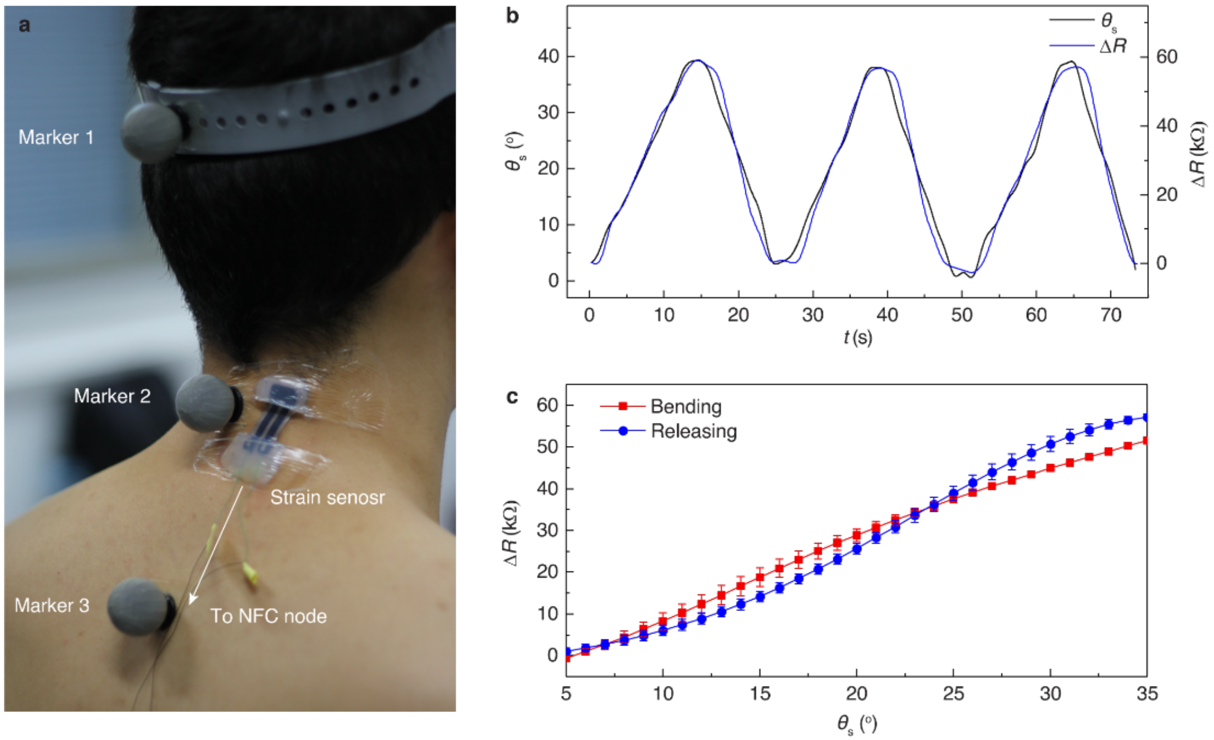

Supplementary Figure 23. Posture monitoring comparison with camera motion capture. a, Photograph of the experimental setup. Three reflective markers along the cervical region of the spine are placed for the camera-based motion capture system (VICON Motion Systems). The strain sensor is attached to the skin next to the center marker, and the NFC circuit placed below the markers to avoid occlusion. **b**, Camera-based measurement of the cervical spine angle θ_s and battery-free sensor measurement *ΔR* during three continuous bend-and-release cycles. **c**, *ΔR* as a function of θ_s during bending and releasing of the cervical spine. Error bars show mean ± s.d. (*n*=3 cycles).

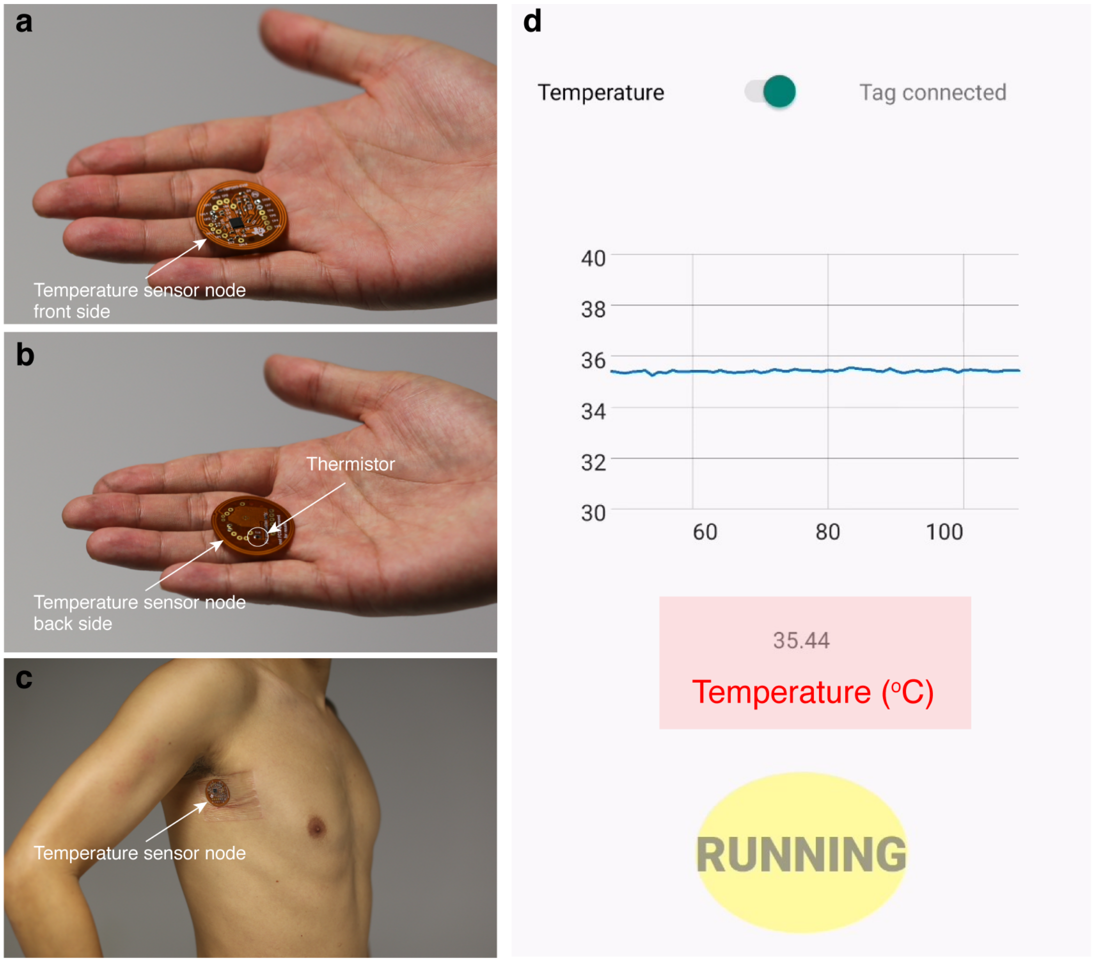

Supplementary Figure 24. Axillary temperature sensing. a-b, Photograph of a commercial temperature senor node shown in front side (**a**) and back side (**b**). The temperature sensor node is wireless and battery-free. The thermistor is located at the back side. **c**, Photograph of the temperature sensor node attached at the armpit of a human subject. **d**, Screenshot of smart-phone application displaying axillary temperature data received from the temperature sensor node shown in (**b**) during the human subject is walking. The sampling rate is 4 Hz.

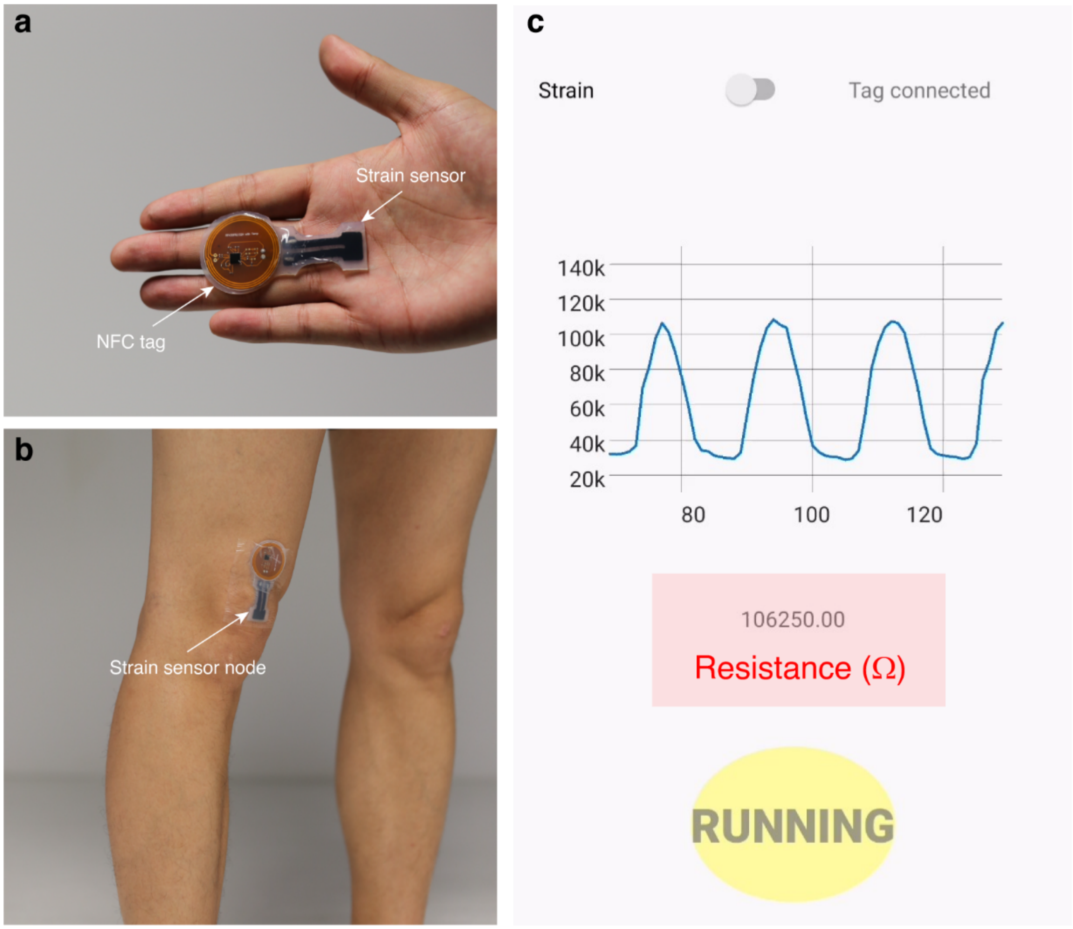

Supplementary Figure 25. Gait monitoring by a strain sensor node. a, Photograph of a strain sensor node. The strain sensor node is wireless and battery-free. **b**, Photograph of the strain sensor node attached at the knee of a human subject. **c**, Screenshot of smart-phone application displaying data received from the strain sensor node shown in (**b**) during the human subject is walking. The sampling rate is 16 Hz.

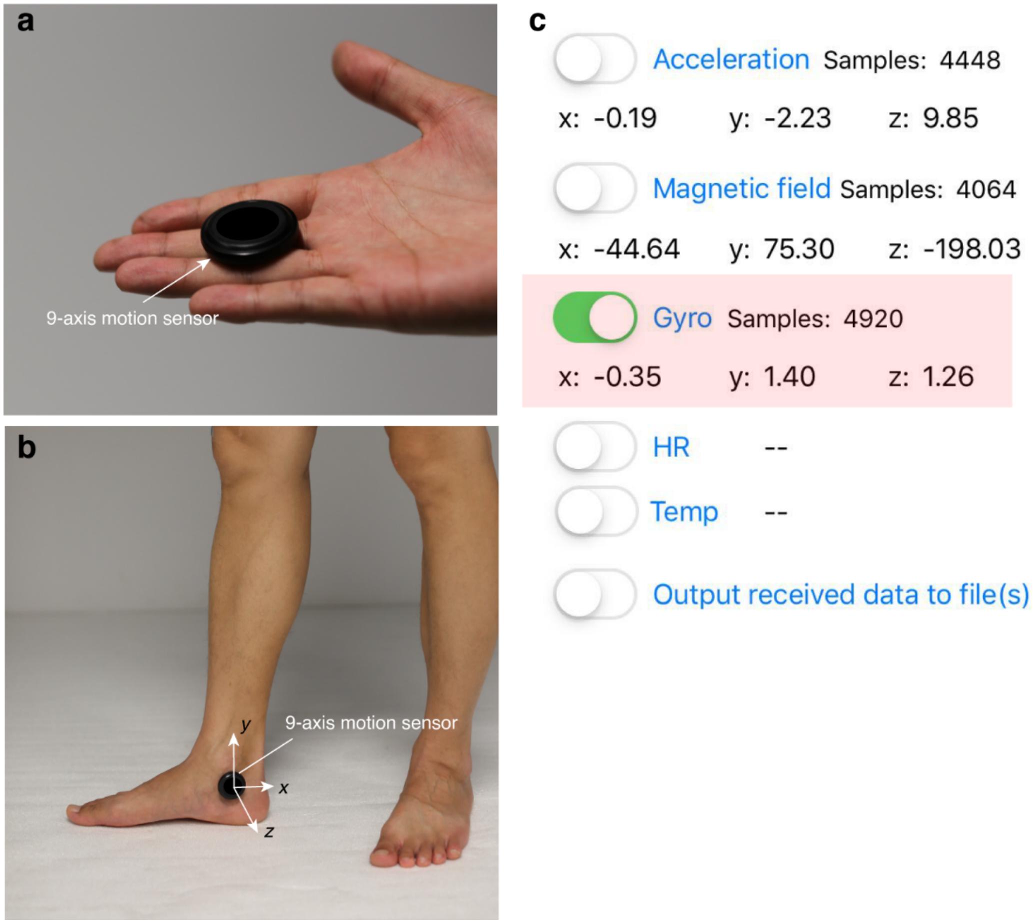

Supplementary Figure 26. Gait monitoring by a gyroscope. a, Photograph of a 9-axis motion sensor (acceleration, gyro, magnetometer) with Bluetooth function. **b**, Photograph of the sensor attached at the ankle of a human subject. **c**, Screenshot of smart-phone application displaying data received from the sensor shown in (**b**) during the human subject is walking. The gyroscope record angular velocity along three directions at a 104 Hz sampling rate. Only the angular velocity along z direction is used for as the reference for gait monitoring.

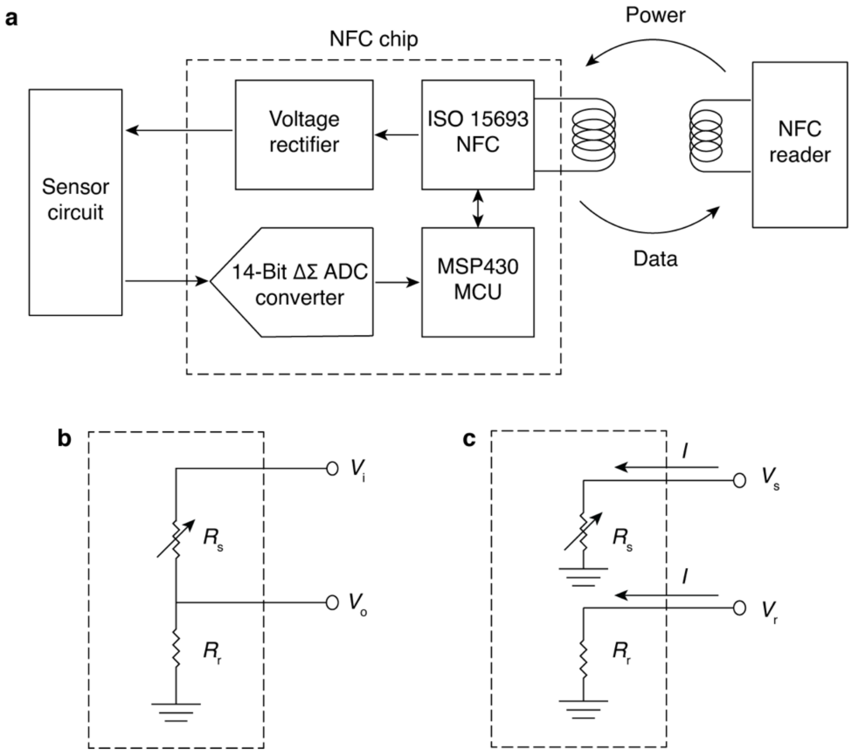

Supplementary Figure 27. Design of NFC sensor node. a, Functional block diagram showing the sensor circuit, NFC chip, and NFC reader module. **b-c**, Schematic of the sensor circuit topology for application requiring high sampling rate (**b**) or high accuracy (**c**).

Supplementary Tables

Supplementary Table 1. Dimensions of the sensor networks interconnected by various near-field-enabled clothing.

Network	Terminal position	Distance-to-hub* (cm)
Powering network (Fig. 2a)	Left sleeve	100
	Right sleeve	100
	Left abdominal	31.5
	Right abdominal	31.5
Spinal posture monitoring network (Fig. 3a)	Top	23.1
	Middle	54.3
	Bottom	57.2
Exercise monitoring network (Fig. 4c,d)	Temperature	30.1
	Gait	38.4

*Distance-to-hub is measured as the interconnect wire length between the inductive pattern of the hub and terminals.
