# Supplementary material for: Wireless battery-free body sensor networks using near-field-enabled clothing
Source: Nat Commun. 2020 Jan 23;11:444. doi: 10.1038/s41467-020-14311-2 (PMC6978350; doi:10.1038/s41467-020-14311-2)
Supplement: Supplementary file 3 — Description of Additional Supplementary Files [file 41467_2020_14311_MOESM3_ESM.pdf]

Description of Additional Supplementary Files

File Name: Supplementary Movie 1
Description: **Wireless powering of LED nodes.** A human subject wears a nearfield-enabled long sleeve shirt with a hub located at the chest and eight terminals distributed around the upper body. A battery-powered NFC reader placed at hub wirelessly powers four LED nodes placed above terminals located on the wrists and abdomen. Operation during human motion demonstrates the robustness of wireless power transfer.

File Name: Supplementary Movie 2
Description: **Multi-node spine posture monitoring with battery-free sensors.** A human subject wears a near-field-enabled shirt with a sensor hub located at the right sleeve and three parallel terminals distributed at cervical, thoracic and lumbar spine. A NFC reader, which supplies power to and collects data from sensor nodes, is placed in a transparent pocket sewed on top of the hub. Three wireless battery-free strain sensor nodes (sensor node 1-3) are attached to the skin under each terminal. The human subject sequentially bends cervical spine, lower back and whole spine, and repeats each posture five times. Data from the three sensor nodes are collected by the reader and displayed on the screen continuously in real-time.

File Name: Supplementary Movie 3
Description: **Axillary temperature sensing and gait monitoring.** A human subject wears near-field-enabled shirt (pant) with a sensor hub located at the right sleeve (back pocket) and a terminal located at armpit (knee). Once a NFC-enabled smartphone is placed on the sensor hub, the battery-free temperature sensor node (strain sensor node) attached to armpit (knee) under the terminal is powered and performs sensing. Data from the sensor nodes are collected by the reader and displayed on the screen in real-time and continuously while the human subject performs walking and running motions.
